# Mammalian tumor-like organs. 1. The role of tumor-like normal organs and atypical tumor organs in the evolution of development (*carcino-evo-devo*)

**DOI:** 10.1186/s13027-021-00412-0

**Published:** 2022-01-10

**Authors:** A. P. Kozlov

**Affiliations:** 1 Vavilov Institute of General Genetics, Russian Academy of Sciences, 3, Gubkina Street, Moscow, Russia 117971; 2 Peter the Great St. Petersburg Polytechnic University, 29, Polytekhnicheskaya Street, St. Petersburg, Russia 195251

**Keywords:** Tumor-like organs, Atypical tumor organs

## Abstract

**Background:**

Earlier I hypothesized that hereditary tumors might participate in the evolution of multicellular organisms. I formulated the hypothesis of evolution by tumor neofunctionalization, which suggested that the evolutionary role of hereditary tumors might consist in supplying evolving multicellular organisms with extra cell masses for the expression of evolutionarily novel genes and the origin of new cell types, tissues, and organs. A new theory—the *carcino-evo-devo* theory—has been developed based on this hypothesis.

**Main text:**

My lab has confirmed several non-trivial predictions of this theory. Another non-trivial prediction is that evolutionarily new organs if they originated from hereditary tumors or tumor-like structures, should recapitulate some tumor features in their development. This paper reviews the tumor-like features of evolutionarily novel organs. It turns out that evolutionarily new organs such as the eutherian placenta, mammary gland, prostate, the infantile human brain, and hoods of goldfishes indeed have many features of tumors. I suggested calling normal organs, which have many tumor features, the tumor-like organs.

**Conclusion:**

Tumor-like organs might originate from hereditary atypical tumor organs and represent the part of *carcino-evo-devo* relationships, i.e., coevolution of normal and neoplastic development. During subsequent evolution, tumor-like organs may lose the features of tumors and the high incidence of cancer and become normal organs without (or with almost no) tumor features.

## Background: the theory of *carcino-evo-devo*

Embryonic and neoplastic development have many common features: intensive cell proliferation, invasiveness, cell migration, the convergence of signaling pathways, important roles of proto-oncogenes and tumor suppressor genes, similarities in gene expression and differentiation, cell adhesion and apoptosis, epithelial-mesenchymal transition, and cell plasticity [1, 2]. Reactivation of embryonic signaling pathways is characteristic of tumors [1, 3, 4]. Tumors may originate from embryonic cells [5–9].

Tumors are widespread and are represented throughout the phylogenetic tree (reviewed in [2, 10, 11]). Proto-oncogenes and tumor suppressor genes are the oldest gene classes [12]. Tumors and cancer-related genes originated during the early period of evolution of multicellular organisms [2, 13, 14].

Many similarities between normal and neoplastic development, and the ancient origin of tumors and proto-oncogenes, assume the long history of the coevolution of normal and neoplastic development. Earlier I hypothesized that hereditary tumors might participate in the evolution of multicellular organisms [15–17]. I formulated the hypothesis of evolution by tumor neofunctionalization (below I will call it "the main hypothesis"), which suggested that the evolutionary role of hereditary tumors might consist in supplying evolving multicellular organisms with extra cells masses for expression of evolutionarily novel genes and the origin of new cell types, tissues and organs [15–17].

Several non-trivial predictions of the main hypothesis have been confirmed in my laboratory. The possibility of selecting tumors for a new function in the organism we confirmed using the novel model of the “hoods” of goldfishes. We performed macroscopic and microscopic studies of adult hoods and the dynamics of the hood growth. We proved histologically that these hoods are benign papillomas [2, 18]. The prediction about the expression of evolutionarily novel genes in tumors was confirmed in many publications from our lab. As a result, a new class of genes—tumor specifically expressed, evolutionarily novel (TSEEN) genes—have been described (reviewed in [19]; see also [12, 20]). The predicted parallel evolution of oncogenes, tumor suppressor genes, and differentiation genes was confirmed using in silico genomic analysis [12]. In the same paper, the predicted correspondence of the number of oncogenes to the number of differentiated cell types was verified [12]. The acquisition of progressive functions, not encountered in fishes, by human orthologs of fish TSEEN genes, was proven using a transgenic zebrafish inducible tumor model [20]. This paper is considered by many specialists as the direct confirmation of the main hypothesis. We also have shown that *the PBOV1* gene, which is overexpressed in breast and prostate cancer, originated de novo in humans and its expression is connected with a favorable clinical outcome of breast cancer [149].

**Fig. 1:**
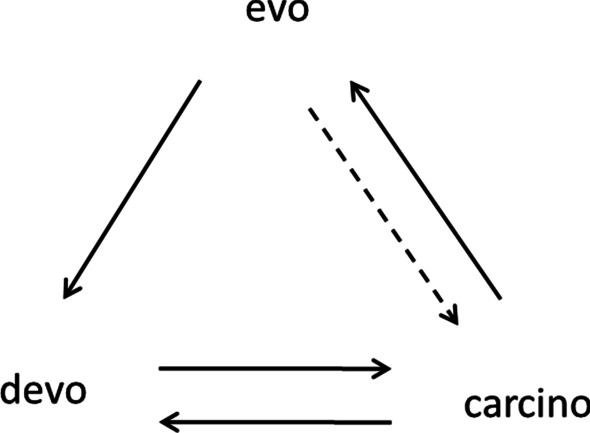
*Carcino-evo-devo* diagram: devo—normal ontogenies. carcino—ontogenies with neoplastic development. evo—progressive evolution of ontogenies. Arrows indicate participation in the corresponding process or essential connections. From A. P. Kozlov (2019) Acta Naturae 11: 65–72, with permission

The accumulating evidence assumed the fundamental nature of the main hypothesis. It was generalized in my book “Evolution by tumor neofunctionalization” [2], which contained more than one thousand references. The book was translated to Russian [21] and Chinese [22]. The main hypothesis started to acquire the shape of the theory. I called it the *carcino-evo-devo* theory. In a recent publication, this theory was further developed [23]. Its relationships with existing biological theories have been examined. The conclusion was reached that *carcino-evo-devo* theory does not contradict the existing biological theories but fills the lacunas between them and explains questions not wholly understood or not explained by current theories. Non-trivial explanations, suggested by the new theory, include the possible role of tumor-bearing organisms as transitional forms in progressive evolution; the role of tumors as the general mechanism to overcome developmental constraints in the origin of major morphological novelties and complex evolutionary innovations; and explanation of the relationships of tumors with embryonic development and *evo-devo*. The diagram describing *carcino-evo-devo* relationships has been suggested (Fig. 1) [23].

Another non-trivial prediction of the new theory is that evolutionarily novel organs if they indeed originated from hereditary tumors or tumor-like structures, should recapitulate some features of tumors in their development. That is why I was looking for the data that might confirm this prediction in the literature, and also performed some experiments in my lab. This paper reviews the evidence that evolutionarily novel organs indeed have many features of tumors that supports the main hypothesis of the possible evolutionary role of tumors and the *carcino-evo-devo* theory [2, 23]. Below I will call evolutionarily new normal organs, which have many tumor features, the tumor-like organs for brevity.

## Main text

### Eutherian placenta, the first identified example of tumor-like organ

Placenta has recurrently originated in different mammalian orders after infection with different retroviruses, which became endogenous to their hosts (reviewed in [2]). The ancestral retrovirus *env* gene has been replaced by new retroviruses and their *env* genes through independent germline infections in different mammalian lineages [24–26]. This is supported by the discovery of retroviral envelope gene capture and syncytin exaptation for placentation in marsupials [27].

*Syncytin* is a domesticated retroviral gene that plays a role in placental biology. Human *syncytin* is the *env* gene of human endogenous retrovirus HERV-W. It is expressed in multinucleated placental syncytiotrophoblast and mediates placental cytotrophoblast fusion (reviewed in [2]).

Eutherian placenta shares many features with tumors. Multinucleated cells and syncytia formation are known features of tumors. Placenta and tumors rely on glycolysis for energy production; the DNA of the placenta demonstrates the genome-wide hypomethylation; some cancer/testis antigens are expressed in the placenta; placental trophoblast is capable to create large ploidies; tumor markers present in the serum of cancer patients include placental proteins; placenta produces angiogenic factors secreted also by tumors (e.g. VEGF), and placenta causes maternal immunosuppression. Parallels between cancer and pregnancy in growth, invasion, and immune modulation have been reviewed, shared characteristics of trophoblast cells and tumor cells, their proliferation and invasion, vasculogenic mimicry and angiogenesis, immunologic similarities of the fetomaternal interface and tumor microenvironment, systemic immune modulation, hypoxia and HIF pathway and other links between cancer and placenta development have been extensively discussed [2, 28–30]. A special conference has been devoted to common features between placental development and cancer growth [29].

The placenta may be considered a regulated tumor [2]. The robust regulation of placental trophoblast by TGF-β efficiently inhibits its malignant properties [31].

### The expression of evolutionarily novel genes in the placenta

The origin of evolutionarily novel genes is connected with the origin of evolutionary innovations and morphological novelties [2, 32]. Domesticated retroviral genes expressed in the placenta were evolutionarily novel to their hosts at the time of infection and acquired new functions in the placenta (syncytins).

There are also other evolutionarily novel genes expressed in the placenta. Transposable elements have donated genes now expressed in the placenta to their ancestral hosts. For example, two Ty3/Gypsy retrotransposon-derived genes, *Peg10* and *Peg11*, are predominantly expressed in the placenta of both humans and mice and are essential for placental development, at early and late stages, respectively (reviewed in [33, 34]).

Many placenta-associated novel genes are found only in certain mammalian species and are not conserved in all Mammalia [33]**.** This is in correspondence with their relatively recent evolutionary origin, i.e., in mammals. For instance, mouse *trophoblast-specific protein* (*Tpbp*) *a* and *b* genes and the related rat gene, *SSP* (*spongiotrophoblast-specific protein*), are specifically expressed in placenta, have been found only in rodents, and are novel. *The placenta-specific protein 1* (*PLAC1*) gene is annotated in the cow, rat, mouse, and human. *Endothelin B receptor*, *Early placenta insulin-like peptide (INSL4)*, *Midline 1*, and *Pleiotrophin* genes are found only in humans and New World monkeys.

### Some novel genes are expressed both in the placenta and in tumors

*PLAC1* gene, annotated in different mammals, was initially thought of as specifically expressed only in the placenta. But later studies [35, 36] have shown that in humans, it is also expressed in testis and various tumors.

LTRs of different HERVs are expressed in the placenta and various tumors [37]. *Syncytin-1* is upregulated in different tumors and participates in their pathogenesis [38–40]. *Early placenta insulin-like peptide*, *INSL4*, found in humans and New World monkeys, is present in breast cancer cells and enhances their invasiveness and motility [41]. The retrotransposon-derived *pleiotrophin* (*PTN*) gene is expressed in both tumors and the placenta. In the placenta, its expression is determined by the LTR promoter [37, 42].

Chorionic gonadotropin (CG) is produced by the placenta to sustain pregnancy [43, 44] and ectopically by a variety of tumors. In early studies, human chorionic gonadotropin has been found in lung carcinoma [45, 46], in other cancers [47], and HeLa cell cultures [48]. CG consists of two subunits, α and β [33, 43, 44]. Subunit α is encoded by a single conservative gene, expressed in the pituitary and placenta, and enters into the composition of all glycoprotein hormones [33, 43]. The different β subunits are encoded by separate genes of the luteinizing hormone (*LHB*)/chorionic gonadotropin (*CGB*) gene cluster. In humans, this cluster contains one *LHB* gene, four β subunit genes (*CGB*, *CGB5*, *CGB8*, and *CGB7*), and two genes (*CGB1* and *CGB2*), which encode novel proteins as a result of frameshifting. All genes of the cluster appear to have originated as a result of duplication of the ancestral *LHB* gene in anthropoid primates. The *LHB*/*CGB* gene cluster is specific to primates. It is an evolutionarily young and unstable genome region [49–52].

The products produced by the human *LHB*/*CGB* gene cluster include the regular CG consisting of two subunits, hyperglycosylated CG, free β subunit, hyperglycosylated free β subunit, and other variants of CG, mostly degradation products, altogether 12 common variants of human CG. A hyperglycosylated free β subunit is produced by almost all nontrophoblastic human malignancies, including cervical cancer, breast, bladder, ovarian, brain, colorectal, uterine, and lung malignancy cell lines [44, 53]. A hyperglycosylated free β subunit may be a molecule with an independent function consisting of the promotion of nontrophoblastic cancer cells' growth and malignancy [44].

Human chorionic gonadotropin beta subunit genes *CGB1* and *CGB2* are transcribed in ovarian cancer tissues [54], in epithelial cancer cell lines [55], as well as in testis [56], pituitary [57], transgenic mouse brain [58], and in the placenta [59, 60]. *CGB1* and *CGB2* have always been considered pseudogenes since the publication of Talmage and co-authors [61], but accumulated expression evidence suggested their functional role. In silico study predicted that as a result of frameshifting, they might encode an utterly novel protein [50]. *CGB1* and *CGB2* genes originated in the lineage-specific to humans and African great apes and are conserved between humans and chimps. In the gorilla, insertion and deletion mutations disrupt the predicted protein. After duplication in the common ancestor of African great apes, *CGB1* and *CGB2* genes may have evolved towards a novel functional gene in humans and chimps and to pseudogenization in gorillas [50].

These many examples of evolutionarily novel genes acting in both placenta and tumors add to the similarity of placenta and tumors and support the hypothesis that the placenta originated from the tumor, induced by oncogenic retrovirus in ancestral eutherian [62–64]. These data also support the broader concept that ancestral hereditary tumors might evolve into functional organs through the expression of evolutionarily novel genes, i.e. evolution by tumor neofunctionalization [2].

Thus, the eutherian placenta represents the first identified example of a tumor-like organ.

## Tumor-like properties of the mammary gland

The mammary gland is an exocrine gland of mammals that produces milk. It constitutes a part of larger structures like breasts and udder.

The mammary gland is a real novel organ in mammals. The mammary gland could be derived from an ancestral apocrine-like gland that was associated with hair follicles [65]. Similarities in signaling and metabolic processes suggest that the mammary gland might also originate from mucous skin glands and the innate immune system as an inflammatory response to tissue damage and infection [66, 67]. According to the other hypothesis, the mammary gland may represent a neomorphic hybrid, a mosaic organ whose evolution involved the incorporation of characteristics encoded in the genome but expressed differently by separate populations of skin glands [68]. The later version of the apocrine-like hypothesis postulates that only mammary ductal tree and secretory tissue evolved from ancestral apocrine-like glands. Additional processes formed the mammary line, placode, bulb, and primary sprout [69].

The mammary gland belongs to mammalian organs with the highest incidence of tumors. Mammary gland tumors are the most common type of tumor in female dogs [70, 71], domestic cats [72], hedgehogs, rats, and mice [73].

Breast cancer is also the most frequent malignant tumor of women in North America and globally [74–76]. According to the Global Burden of Disease (GBD) 2015 study, breast cancer is the most common cancer type and the leading cause of cancer deaths in females [77].

The evolutionary novelty of the mammary gland may be a reason for the higher incidence of breast cancer in humans as compared to cancer incidences in evolutionarily older organs [78].

The mammary gland is the organ developing predominantly after birth. The developing mammary gland demonstrates many of the properties associated with tumors, such as invasion, re-initiation of cell proliferation, resistance to apoptosis, an essential role of stromal cells, and angiogenesis. Terminal end buds (TEBs), a rapidly proliferating mass of epithelial cells, invade into stromal tissue, much like a solid tumor [79].

During its developmental cycle, the mammary gland displays both invasive growth and regression. During mammary gland development, the mammary epithelium invades the fat pad and forms a small, branched ductal network. The epithelium does not fill the fat pad until the release of ovarian hormones at puberty. After that, TEBs form, and the ducts invade, branch, and fill the pad. During pregnancy, epithelium proliferates, ducts form side branches, and alveolar structures form and differentiate. The epithelium expands almost to fill the mammary gland and becomes secretory. At the same time, large fat cells dedifferentiate into small pre-adipocytes. During involution, the secretory epithelium dies by apoptosis, and the fat cells re-differentiate [79].

The epithelium of the terminal end buds of the developing mammary gland resembles the epithelium of mammary tumors. The term “morphogenetically active epithelial state” was suggested to describe the mammary epithelium during morphogenesis [80]. A mammary stem cell population has similarities in expression profiles to human breast cancer (basal-like and Her2^+^ intrinsic breast cancer subtypes) [81].

Epithelial plasticity and invasive properties are critical during branching morphogenesis in the mammary gland. On the other hand, epithelial-mesenchymal transition (EMT) and epithelial plasticity contribute to tumor progression. Thus, there are similarities between developmental and oncogenic EMT in the mammary gland [3].

### Similarities in immune regulation of mammary gland development and tumorigenesis

Innate immune cells (mast cells, eosinophils, and macrophages) play roles in terminal end bud elongation and branching morphogenesis during postnatal mammary gland organogenesis [82–86]. Macrophages have a role in regulating epithelial cell death during mammary gland involution [87, 88]. During tumorigenesis, similar innate immune cells are recruited. Mast cells, macrophages, and neutrophils promote tumor progression by stimulating angiogenesis, suppressing antitumor immunity, and enhancing tumor cell migration and metastasis [86]. Abrogation of TGF-β signaling in mammary carcinomas recruits Gr-1 + CD11b + myeloid cells that promote metastasis. The authors point out that innate immune cells may not play an immunological role in development because there is no pathogens present. Instead, they may be trophic to developing tissue, enhance rates of epithelial growth and invasion, and influence the complexity of the ductal structures [86, 89].

The most striking is that the adaptive immune system also contributes locally to postnatal mammary organogenesis. Antigen-mediated interactions between mammary CD11c + antigen-presenting cells and IFNγ-producing CD4 + T helper 1 cells provide signals that negatively regulate ductal invasion. IFNγ mediates the inhibitory effect of CD4 + Th1 cells on mammary organogenesis by affecting luminal differentiation. The nature of the antigen(s) involved is unknown [90]. During tumorigenesis, there is a much higher engagement of the acquired immune response. The presence of many acquired immune cells in tumors suggests the recognition of new "foreign" tumor antigens or the extensive tissue damage caused by tumor growth. CD8+, T_H_1, and NK cells protect against tumor development and progression. However, B cells, activation of humoral immunity, and infiltration of T_H_2 cells, as well as innate inflammatory cells, may promote tumor progression. Thus innate and acquired immune cells are in the dynamics. The regulatory functions of the immune system are conferred on the epithelial tumors in a fashion that mimics development [86].

### Signaling pathways

The reactivation of developmental pathways in breast and other cancers contributes to tumor progression. Developmental EMT regulators, including Snail/Slug, Twist, Six1, and Crypto, are misexpressed in breast cancer [3]. Three major stem cell signaling pathways (Notch, Wnt, and Hedgehog) and other critical cellular signaling pathways (estrogen receptor, PI3K, MAPK, JAK/STAT, NFκB, and TGF-β) participate both in normal mammary gland development and in breast cancer and cancer stem cells [91, 92].

### TGF-β regulation

Mammary epithelial cells are sensitive to TGF-β. During mammary gland development, stromal TGF-β inhibits proliferation and morphogenesis. On the contrary, most breast cancer cells are not responsive to the cytostatic action of TGF-β. Although mutations in TGF-β receptor genes are infrequent in human breast cancers, there is compelling evidence for impairment of TGF-β signaling in this disease [93]. The paradox of TGF-β is that it suppresses the proliferation of normal breast epithelial cells, but converts to a promoter during cancer development [94, 95]. There is clinical evidence that TGF-β acts as a tumor-derived immunosuppressor, an inducer of tumor mitogens, a promoter of carcinoma invasion, and a trigger of prometastatic cytokine secretion [96]. TGF-β also drives the acquisition of invasive behaviors in cancer cells undergoing an epithelial-mesenchymal transition (EMT) [95, 97, 98].

### Hormone action

Two-thirds of all breast cancers are hormone-dependent. Breast cancer is classified based on the presence or absence of the estrogen receptor, progesterone receptor, and human epidermal growth factor receptor-2 (HER2). Hormones influence the course of a disease by affecting angiogenesis, stemness of breast cancer stem cells, inducing chemoresistance, and favoring metastatic growth. The same hormones control postnatal mammary gland development during puberty, pregnancy, lactation, and involution. Estrogens, progesterone, and prolactin act sequentially on the mammary epithelium in synergy with corticosteroids and the presence of the growth hormone. Sequential activation of hormone signaling in the mammary epithelium is required for the progression of morphogenesis [99, 100].

### Breast cancer susceptibility genes *BRCA1* and *BRCA2* are tumor suppressor genes and participate in normal development

Hereditary breast cancers due to germline mutations in the breast cancer susceptibility genes *BRCA1* and *BRCA2* are very common. *BRCA* genes are tumor suppressor genes, and germline mutations destroying their functions also cause ovarian cancer and other malignancies [101–103]. The molecular functions of *BRCA* genes are connected with genome stability. The loss of these functions due to mutations causes genome instability and leads to oncogenic transformation. *BRCA* genes have also other tumor-related functions, the most important for the present consideration is participation in the regulation of cancer stem cells [104].

On the other hand, *BRCA* genes participate in normal development. *Brca1* and *Brca2* genes are required for embryonic cellular proliferation and differentiation in the mammary gland and other tissues in the mouse [105, 106]. *BRCA1* is expressed by embryonic and adult neural stem cells and is involved in neural stem cell proliferation in rats [107]. In primates, *BRCA1* evolves rapidly under positive selection and has implications both for cancer predisposition and brain development [108–110].

### Intermediary conditions

So-called "responsive" spontaneous mammary tumors in mice grow during pregnancy, reach a peak before parturition, and regress after that [111]. The mammary gland tumors of rats are mostly benign fibroadenomas [73]. In dogs and cats, benign mammary tumors are challenging to differentiate histologically from the physiological hyperplasia of the mammary gland. Differential diagnosis between complex adenoma and complex adenocarcinoma is also tricky [112]. In women, the vast majority of the lesions that occur in the breast are benign [113]. Benign breast disease is a broad category of diagnoses (including developmental abnormalities, inflammatory lesions, epithelial and stromal proliferation, and neoplasms) with a variable degree of increased risk of developing breast cancer [114]. Disordered development may result in tumor-like lesions such as hamartomas, pseudoangiomatous stromal hyperplasia, and gynecomastia [115]. Compared to women with normal pathology or non-proliferative disease, women with a proliferative disease without atypia have a modestly increased risk of breast cancer. In contrast, women with atypical hyperplasia have a substantially increased risk [116]. Breast fibroadenomas are not associated with increased breast cancer risk in African American women [117]. Lobular and ductal carcinomas in situ are not considered to be obligatory precursors of invasive breast cancer [113, 118].

### Milk and mammary genes

Milk originated as a glandular skin secretion in synapsids, the ancestors of mammals. That is why the mammary gland coopted signaling pathways and genes for secretory products from earlier integumentary structures [66, 119]. Milk has both protective and nutritional roles for mammalian neonates [66, 67]. Xanthine oxidoreductase (XOR) and lysozyme are two important antimicrobial enzymes of the innate immune system. Due to gene sharing, XOR is also required for the secretion of milk fat globules [120]. α-Lactalbumin, a whey protein and a subunit of the lactose synthase heterodimer, evolved from a gene duplicate of lysozyme [66]. Besides participating in lactose synthesis, alpha-lactalbumin functions as an apoptotic factor that kills tumor cells [121] and regulates the involution of the mammary gland [122].

Caseins evolved in the mammalian lineage. In milk, casein and calcium phosphate combine into casein micelle. Caseins show high substitution rates and belong to the secretory calcium-binding phosphoprotein gene family that arose by gene duplication [123].

The most divergent proteins of milk are associated with nutritional and immunological components of milk, and the most conserved proteins are associated with the secretory process.

More milk and mammary genes are present in all mammals, and more duplicated after common ancestor with platypus than other genes of the mammalian genome [124]. A recent study of novel genes in placental mammals discovered novel genes expressed in breast tissue [125].

The opossum genome's sequencing revealed that a considerable proportion of eutherian non-coding elements originated after the divergence of Eutheria and Methatheria. Part of these eutherian-specific non-coding sequences originated from transposons [126]. Endogenous retroviruses expressed in the mammary gland are also evolutionarily novel to mammals.

### Mouse mammary tumor virus (MMTV) and other viruses

MMTV causes most of the mammary tumors in mice [73]. MMTV exists as an exogenous infectious virus, and as an endogenous virus. Both can cause mammary tumors when the provirus integrates into the mammary epithelial and lymphoid cell genome and activates cellular oncogene expression [127]. MMTV first infected *Mus* germline approximately 10 million years ago, after their speciation from rats [128]. MMTV infection may have a dual effect: physiological increase of lobuloalveolar differentiation and pathological tumorigenic activity. These are separate activities that use different pathways [186]. The physiological activity of MMTV could participate in the evolution of the development of the mammary gland. The experiments can be designed to further study this involvement.

Many species, including humans, contain endogenous retrovirus sequences related to MMTV. The most recently integrated HERV provirus, HERV-K, belongs to a subgroup most highly related to MMTV [129]. The accumulating evidence suggests that exogenous MMTV-like virus and HERV-K, individually or in concert, can cause mammary tumors in humans. Other viruses (bovine leukemia virus, human papillomaviruses, and Epstein-Barr virus) may also have a role [127, 130–135].

We currently study the phenomenon of TSEEN genes with human endogenous retroviruses (HERVs). It is known that different families of HERVs infected human ancestors during different phylogenetic periods. We suggested that evolutionarily youngest HERV-K HML-2 sequences should have higher expression levels in tumors and evolutionarily younger organs, e.g. mammalian mammary gland. We analyzed the expression of twelve HERV-K HML-2 sequences located on human X-chromosome and found that these sequences are expressed significantly higher in tumors (lung small cell carcinoma, colon cancer, and acute myeloid leukemia) than in corresponding normal tissues (lung, colon, and lymphocytes). However, there was no difference in expression levels of these sequences between normal mammary gland and breast cancer cells, which supports the tumor-like nature of the mammary gland [185].

### Coevolution of the mammary gland with the placenta

The evolution of the mammary gland and placenta culminates in Eutherians, where the impact of placentation and lactation in rearing young animals is approximately equal. In marsupials, the imperfect short-lived placenta forms late in pregnancy (and in a different way), but lactation is extended. The mammary gland of marsupials performs many of the functions of the eutherian placenta. Some genes expressed in the eutherian placenta are expressed during lactation in marsupials [136, 137]. Placental hormones are critical regulators of mammary gland development and lactation [138]. Syncytin participates in breast cancer-endothelial cell fusions [38]. PLAC1 (placenta-specific protein 1) is expressed in breast cancer and could be a serum biomarker for breast cancer [139].

## The prostate gland is a tumor-like organ

The prostate gland has many biological similarities with the mammary gland. Like the mammary gland, the prostate originated in placental mammals [140]. (The prostate glands of male marsupials are disseminated [141]). In the course of evolution, both glands developed many similarities in physiology, endocrinology, and oncology. Similarities of prostate and breast cancer are outstanding and include common epidemiological, biochemical, and genetic features [142–144]. Both types of cancer are hormone-dependent [144]. Germline mutations in breast cancer predisposition genes 1/2 (BRCA1/2), especially in the BRCA2 gene, are predictive factors for prostate cancer also [145, 146]. On the other hand, prostate-specific antigen (PSA) is found in normal breast tissues and fluids, in breast tumors, and in benign breast disease [147]. *PBOV1* gene is overexpressed in breast and prostate cancer [148]. This gene originated de novo in humans; its expression is connected with a favorable clinical outcome of breast cancer [149]. On the other hand, *PBOV1* rs6927706 polymorphism is associated with an increased risk of developing breast cancer [150].

Like the mammary gland, the prostate involutes upon deprivation of hormonal factors, e.g., upon castration [151].

Similar to the mammary gland, the prostate gland demonstrates the correlation between evolutionary novelty and the highest incidence of cancer [78]. The GBD 2015 study reported that for men, the most common type of cancer globally was prostate cancer [77]. In the US, one in eight men will be diagnosed with prostate cancer during their lifetime. Prostate cancer is the leading cancer type for new cancer cases, and the second leading cause of cancer lethality in men [152]. Age-specific prostate cancer prevalence, determined by autopsy studies, reaches 59% by age > 79 years [153]. In older men, the prevalence of benign prostatic hyperplasia may reach 100% (reviewed in [154]), which produces the impression that the prostate is a benign tumor slowly growing throughout the life of an individual.

The prostate also has similarities with the placenta. PSA is synthesized and excreted by the placenta [155]. On the other hand, placenta-specific protein 1 (PLAC1) is expressed in prostate adenocarcinoma [156].

Like the placenta and mammary gland, the prostate has a regulated invasion stage in its organogenesis. At the earliest stages of prostate development, prostate epithelial buds invade into surrounding mesenchyme. Genes expressed during prostate cancer progression overlap with genes expressed at the most initial stages of prostate development [157].

This evidence indicates the tumor-like nature of the prostate gland. Recapitulation of neoplastic features at the earliest stage of prostate development, when its identity is first becoming established, points at the possible origin of a prostate from the tumor.

Prostate accumulates the amount of zinc almost an order of magnitude higher than other tissues. The high amounts of zinc and citric acid in prostatic fluid (their metabolism is linked to the prostatic gland) are important for the functioning of spermatozoa. In prostate tumors, the concentration of zinc is lower due to the downregulation of zinc transporters ZIP1, ZIP2, and ZIP3 [181–183]. We may guess that the primary adaptation provided by the benign tumor ancestor of the prostate could be an initial accumulation of zinc, which was selected for in evolving prostate organs because it supported the viability of spermatozoa. Downregulation of zinc concentration in prostate tumors may recapitulate the initial evolutionary condition of the ancestor tumors.

Hereditary/familial prostate cancer is described [158, 159]. Prostate cancer predisposition genes include *ATM, BRCA1, BRCA2, CHEK2, EPCAM, HOXB13, MLH1, MSH2, MSH6, NBN, PALB2, PMS2, RAD51D,* and *TP53* genes. *BRCA2, ATM, CHEK2,* and *HOXB13* mutate more often. The multigene panel is suggested as the primary germline testing for hereditary prostate cancer [184].

## Human brain recapitulates many features of tumors

The human brain, the most recently evolved organ, has many features recapitulating those of tumors. Besides a disproportional increase in size, these features include production of excessive neurons during development; aneuploidy connected with recombination-related genes; many additional copies of L1 transposable elements; genetic mosaicism; high level of gene expression; the involvement of many proto-oncogenes and tumor suppressor genes in brain evolution and development (reviewed in [2]). Evolutionarily novel genes, including cancer/testis/brain genes, are expressed in the brain [2, 160].

The pattern of directional and accelerating evolution towards larger brain size has been described within hominins. The human brain is exceptionally large for primates, "238% larger than the size expected for a primate of similar body mass and phylogenetic position" [175].

During normal development, prolongation of the high prenatal rate of brain growth into early childhood results in a human-size brain, which is much larger than the monkey’s brain. Studies of the ontogenic allometry have shown that prenatal brain-body curves for humans and monkeys are identical, but humans extend their curve into postnatal ontogeny, until two years after birth (reviewed in [2]). That is probably why the human brain demonstrates more of its tumor-like nature during childhood and infancy. Brain cancers are the most common type of solid organ tumor in children and are the leading cause of cancer death in children [161–163].

The theory of *carcino-evo-devo* suggests that it was a heritable benign tumor-like process that supplied evolving human ancestors with additional cell masses for brain evolution. Indeed, expansion of the human cerebral cortex may be a result of selection for tumor growth connected with the human-specific loss of tumor suppressor gene *GADD45G* enhancer [164]. Tumor suppressor gene *BRCA1* (breast cancer susceptibility gene 1) is rapidly evolving under positive selection in primates and humans and participated in the evolution of brain size in humans [2, 108–110]. Brain tumors possess mechanisms of neural plasticity. Many gliomas molecularly and phenotypically resemble oligodendrocyte precursor cells. Gliomas functionally integrate into electrically active neural circuits through neuron-to-glioma signaling [165].

## Pseudodiseases and tumor-like conditions in other organs

Infantile in situ neuroblastomas, detected by screening, represent an interesting example of tumors fundamentally different from symptomatic tumors. Their natural history is mostly unknown. Neuroblastomas may regress or differentiate into benign cells in older children.

Infant screening programs for neuroblastoma demonstrated increasing incidence rates of early-stage tumors. Still, they did not show any increase in more advanced tumors and deaths due to neuroblastoma (reviewed in [2]). It was suggested to call such lesions the “pseudodisease” [166, 167].

Tumor-like conditions exist in different normal organs. Tumor-like conditions can be defined as conditions that macroscopically and/or microscopically may appear as neoplasms but are not truly neoplastic [176]. Many organs (bones, pleura, lung, heart, brain, etc.) have tumor-like conditions which complicate the diagnostics of the tumors. Tumor-like malformations (e.g. hamartomas and choristomas) occur anywhere in the body and may be confused with true neoplasms [177, 178]. Breast lumps may be non-neoplastic, benign, and malignant. Tumor-like conditions of the mammary gland discussed above may never progress to cancer. The borders between tumor-like conditions, benign and malignant tumor processes in the mammary gland are difficult to draw, and the prognosis is difficult to make.

The borderline tumors (tumors of low malignant potential) may be associated with tumor-like conditions. For example, borderline ovarian tumors may be related to ovarian endometriosis—the tumor-like condition of endometrial cells growing outside the uterus in about 10% of reproductive-age women [179, 180].

The existing evidence produces the impression that normal organs are not fixed and stable entities, but fluctuating and relatively unstable in terms of their cellular composition and proliferative processes, sometimes resembling tumors. This may reflect the role of hereditary tumors in their origin, which may consist in providing cellular material for the natural selection of new or improved organs.

## Tumor-like organs in other animals

Breeders have selected varieties of goldfish which develop hoods (Oranda and Redcap Oranda, Lion head, Ranchu) during the last several hundred years. Thus, the "hoods" of goldfishes are less than one thousand years old and may be considered as evolutionarily new organs.

We studied the morphology and dynamics of hoods growth in goldfishes [2, 18]. We performed macroscopic and microscopic studies of adult hoods and the dynamics of the hood growth. A population of baby fishes obtained by hybridization of Oranda and Fantail goldfishes was observed for two years. Individual fishes were periodically taken for the histological study of the head skin. We proved histologically that these hoods are benign papillomas [2, 18].

The hoods differ from malformations by progressive changes of macroscopic and microscopic features. From reactive proliferates, the hoods differ by the absence of inflammation and no tendency to regression. The hoods do not have the characters of malignancy. Thereby the most likely conclusion would be that the goldfish hoods represent genetically determined benign tumors [2, 18].

That is, benign tumors were artificially selected for. As a result of this selection, a new organ—the hood—originated. This is the first example of artificial selection of benign tumors described in the literature [2, 18].

The symmetrical shape of the hoods and their benign nature make them similar to organs. The progressive character of their growth makes them similar to tumors. That is why hoods of goldfishes may be considered as tumor-like organs.

## Tumor-like features of evolutionarily novel organs suggest their origin from, or with the help of, hereditary tumors

Tumor-like organs discussed in this paper are evolutionarily young or novel organs. The "hoods" of goldfishes are a few hundred years old. Placenta, mammary gland, and prostate are characteristic traits of mammals, even though their ancestral forms originated somewhat earlier. The human neocortex is human-specific. Thus, tumor-like properties of the discussed organs may be connected with their evolutionary novelty, as predicted by the main hypothesis and *carcino-evo-devo* theory [2, 23].

We see that tumor-like organs have many features of tumors. Tumor-like properties of the placenta and mammary gland are so remarkable that researchers use the placenta as the model of tumor progression [31], and the mammary gland involution as a model of tumor regression and the other complicated features of tumors [168]. Prostate at the early stage of development was considered as a model system for the investigation of genes that drive prostate cancer [157].

The critical feature of the mammary gland and prostate is the high rate of cancer incidence. Brain cancers are the most common type of solid organ cancer in children. Earlier it was shown that the evolutionary novelty of organs correlates with cancer rates in humans [78]. Davies asked a question, “Why should this be?” but didn't answer. He dismissed the argument that selection pressure had less time to reduce neoplastic tendencies because this would mean that the evolution of new organs would 'reset' the risk of neoplasia. Davies saw no reason for that.

But the theory of *carcino-evo-devo* [2, 23] provides such a reason. This theory suggests that hereditary tumors at the earlier stages of progression might participate in the origin of new cell types, tissues, and organs through the expression of evolutionarily novel genes in tumor cells. Tumor specifically expressed, evolutionarily novel genes have been described in my lab [12, 19, 20, 149].

According to *carcino-evo-devo* theory, new organs may originate from hereditary tumors, as shown for “hoods” of goldfishes [2, 18], and for the placenta [62–64]. Hereditary tumors may also participate in the evolution of existing organs, as in the case of the origin of symbiovilly in the stomach of voles [169] and neocortex [164], and in the origin of new cell types, as in the case of the origin of macromelanophores from melanoma cells in swordtails (reviewed in [2]). If tumor neofunctionalization indeed took place in evolution, it would result in similarities of normal and neoplastic development, and features of tumors and higher cancer rates in evolutionarily new organs, like in mammary gland and prostate.

Thus, the selection pressure indeed had less time to reduce neoplastic tendencies in novel organs, as follows from the hypothesis of evolution by tumor neofunctionalization. I would agree with Davies that recently evolved differentiation states could be less stable. As I discussed earlier [2], there should be a positive selection for reinforcement of the evolutionarily novel functions and regulatory feedbacks, as in the case of evolutionarily novel genes that encode evolutionary novelties and morphological innovations. So, we could anticipate the dynamic picture and the whole gradient of relatively unstable transitionary structures (tumor-like organs), leading to the origin of evolutionarily novel organs. We also should look more carefully for tumor-like transitionary structures in paleontological records.

The ancient condition of the most invasive hemochorial placenta [170, 171] and participation of the adaptive immunity in postnatal mammary organogenesis [90] are difficult to explain otherwise than by suggesting the tumor nature of ancestral organs. The mosaicism of the mammary gland, the evolution of which involved the incorporation of characteristics expressed initially by separate populations of skin glands [68], is explained by the "tumors as search engine” concept [2, 23] if we suggest the tumor nature of the ancestral mammary gland.

Recent hypotheses suggest that cancer development may be promoted by reactivation of placentation programs and that tumors recapitulate features of the placenta [30, 172]. I offer a different scenario: tumor-like properties of the placenta and other organs discussed in this paper may be a recapitulation of their origin from ancestral hereditary tumors.

## Tumor-like organs might originate from hereditary atypical tumor organs

Solid tumors are not amorphous masses of cells but have features of normal organs. Solid tumors have parenchyma and stroma. Parenchyma consists of a hierarchy of cell types at different stages of differentiation, similar to that in normal organs, i.e. undifferentiated cancer stem cells (CSCs), transit-amplifying cells, and differentiated cells. The stroma consists of connective tissue, blood vessels, and accessory cells. Although differentiation of tumor cells is not perfect and regulatory feedback loops are weak or non-existent, the concept of tumors as atypical organs is well spread among oncologists (reviewed in [2, 173]).

Many tumors are inherited. Hereditary cancer syndromes are even more frequent than non-cancer genetic syndromes (reviewed in 2, 174]).

The main hypothesis suggests that hereditary atypical tumor organs could be used by natural selection for the origin of new organs, or for the evolution of existing organs. This might happen if ancestral hereditary tumors acquired regulated functions, and tumor-bearing organisms survived long enough to leave a progeny. Thus, hereditary atypical tumor organs may be the initial stage in the evolution of novel tumor-like organs.

## Tumor-like organs, atypical tumor organs, and the theory of *carcino-evo-devo*

**Fig. 2:**
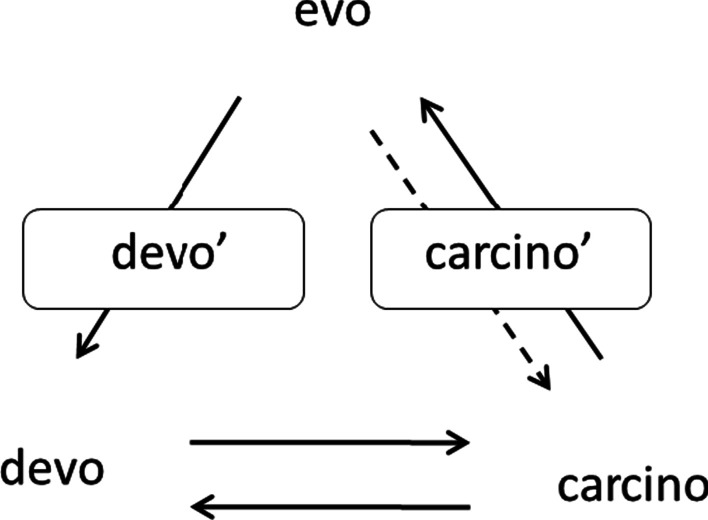
*Carcino-evo-devo* diagram with tumor-like organs and atypical tumor organs: devo—normal ontogenies. carcino—ontogenies with neoplastic development. evo—progressive evolution of ontogenies. devo’—tumor-like organs. carcino’—atypical tumor organs

Tumor-like organs and atypical tumor organs may occupy intermediate, transitory positions on *the carcino-evo-devo* diagram (Fig. 2). Normal tumor-like organs, from one side, and tumors as atypical organs, from the other side, thus help to fill the gaps in the description of the origin of evolutionarily new organs from hereditary tumors.

**Fig. 3:**
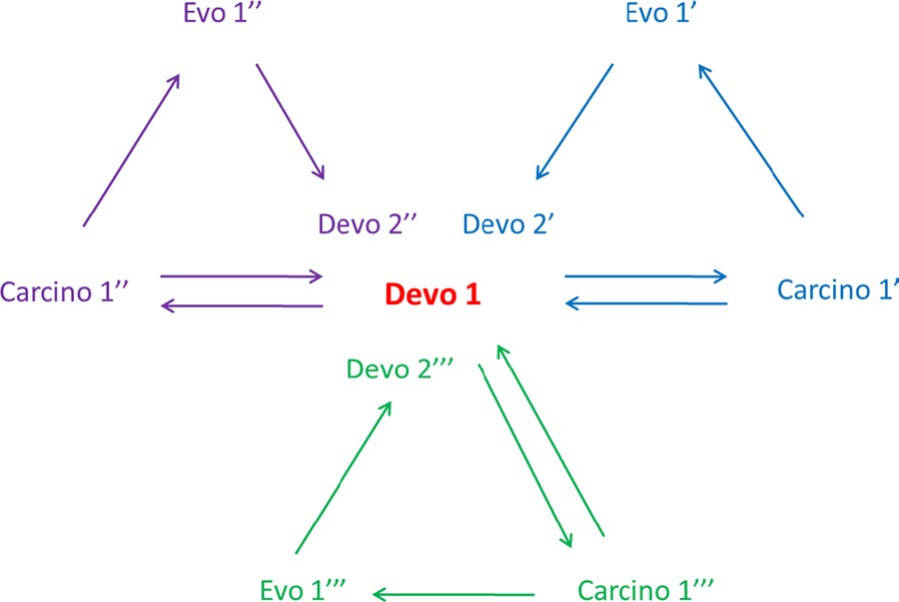
*Carcino-evo-devo* diagram illustrating the origin of the eutherian placenta, mammary gland, and prostate in ancestral eutherians

The origin of the eutherian placenta, mammary gland, and prostate in ancestral eutherians may be represented in the following way (Fig. 3). As follows from Fig. 3, the ancestral ontogenesis (Devo 1) has evolved three additional processes of organogenesis (Devo 2’, Devo 2’’ and Devo 2″’) through the participation of three different hereditary tumors/atypical tumor organs (Carcino 1’, Carcino 1’’ and Carcino 1″’).

**Fig. 4:**
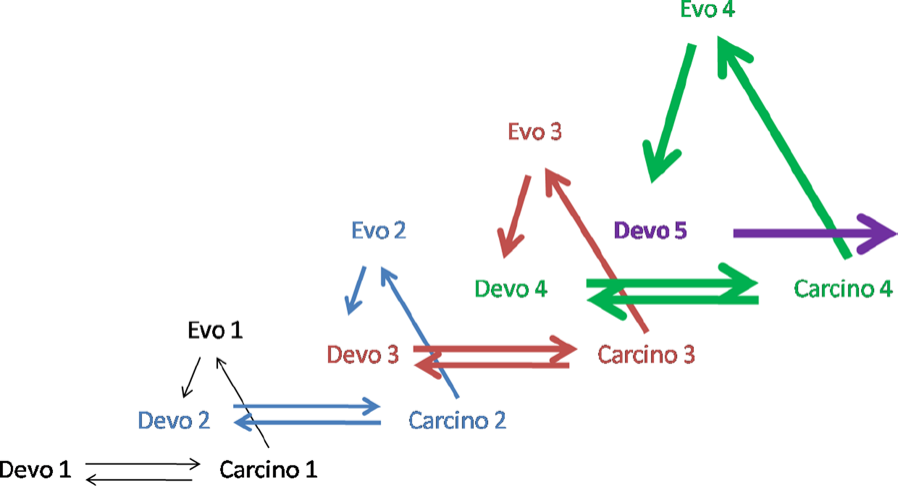
*Carcino-evo-devo* diagram showing several successive steps in the progressive evolution of ontogenies. From A. P. Kozlov (2019) Acta Naturae 11: 65–72, with permission

Devo 2, which includes Devo 1 and three novel organs (Devo 2 = Devo 1 + Devo2’ + Devo 2″ + Devo 2’’’), can further evolve with the origin of younger organs like a human brain with its neocortex (e.g., Devo 5 at Fig. 4) with the help of the other hereditary tumors/atypical tumor organs (Carcino 4 at Fig. 4).

Thus, the concepts of tumor-like normal organs and tumors as atypical organs help to understand better the *carcino-evo-devo* relationships and the role of hereditary tumors as the transitory condition in the evolution of normal development.

## Conclusion

We see that evolutionarily novel organs such as the eutherian placenta, mammary gland, prostate, infantile human brain, and hoods of goldfishes have many features of tumors and may be considered as normal but tumor-like organs. Tumor-like organs might originate from hereditary tumors and atypical tumor organs and represent the part of *carcino-evo-devo* relationships, i.e., coevolution of normal and neoplastic development, and involvement of hereditary tumors in the evolution of development. During subsequent evolution, tumor-like organs may lose the features of tumors and the high incidence of cancer and become normal organs without (or with almost no) tumor features. However, the proneness of different normal organs to cancer development, although with varying rates of incidence, is the tumor feature. According to the *carcino-evo-devo* theory, this may be a recapitulation of the origin of new organs from the ancestral hereditary tumors.

## Data Availability

Not applicable.

## Abbreviations

GADD45G: Growth arrest and DNA-damage-inducible protein
HERVs: Human Endogenous Retroviruses
HERV-K: The human endogenous retrovirus type K
HIF: Hypoxia-inducible factors
JAK/STAT: The Janus kinase/signal transducers and activators of transcription
LTRs: Long terminal repeats
MAPK: Mitogen-activated protein kinase
NFkB: Nuclear factor kappa-light-chain-enhancer of activated B cells
PI3Ks: Phosphoinositide 3-kinases
TGFb: Transforming growth factor beta
VEGF: Vascular endothelial growth factor
